# Effect of the COVID-19 Pandemic on Radical Prostatectomy: A Turkish Multicenter Study

**DOI:** 10.5152/tud.2022.22130

**Published:** 2022-09-01

**Authors:** Abdullah Gurel, Burhan Baylan, Ibrahim Keles, Arif Demirbas, Mustafa Karalar, Berkay Eren, Ata Ozen, Unal Oztekin, Can Benlioglu, Ahmet Emin Dogan, Mehmet Altan, Erol Ersekerci, Eren Cengiz, Mert Ali Karadag, Ahmet Yaser Müslümanoğlu

**Affiliations:** 1 Department of Urology, Afyon Health Sciences University Faculty of Medicine, Afyon, Turkey; 2 Department of Urology, Eskisehir Osmangazi University Faculty of Medicine, Eskisehir, Turkey; 3 Department of Urology, Kayseri System Hospital, Kayseri, Turkey; 4 Department of Urology, Adıyaman University Faculty of Medicine, Adıyaman, Turkey; 5 Health Sciences University, Dışkapı Yıldırım Beyazıt Training and Research Hospital, Urology Clinic, Ankara, Turkey; 6 Department of Urology, Kırşehir Ahi Evran University Faculty of Medicine, Kırşehir, Turkey; 7 Kayseri City Education and Research Hospital, Kayseri, Turkey; 8 Department of Urology, İstanbul Bağcılar Training and Research Hospital, Turkey

**Keywords:** COVID-19, prostate cancer, radical prostatectomy, upgrading

## Abstract

**Objective:**

The present study examines the effects of the coronavirus disease 2019 pandemic on radical prostatectomy performed as part of localized prostate cancer treatment in Turkey.

**Material and methods:**

A retrospective analysis was made of the data of 176 patients from 8 centers in Turkey who underwent radical prostatectomy due to localized prostate cancer over the 2 years spanning March 1, 2019, to February 28, 2021. Within this timeframe, March 1, 2019, to February 28, 2020, was denoted the 1-year pre-coronavirus disease 2019 period, while March 1, 2020, to February 28, 2021, was denoted the 1-year coronavirus disease 2019 period. An analysis was made of whether there was a difference in the number of radical prostatectomies performed for prostate cancer, the time from biopsy to operation, and the biopsy and radical prostatectomy pathology between the 2 periods.

**Results:**

It was found that the number of radical prostatectomies performed for localized prostate cancer during the coronavirus disease 2019 pandemic was statistically and highly significantly fewer than in the pre-coronavirus disease 2019 period (*P*  < .001). The patients diagnosed with Gleason 3 + 3 (low risk) prostate cancer were statistically significantly fewer in number in the coronavirus disease 2019 period (*P*  < .001). The pathological Gleason score was upgrading than the biopsy Gleason score in all patients who underwent in both periods (*P*  < .001). When the periods were compared, the pathological involvement determined by lymph node dissection performed during radical prostatectomy was found to be decreased in the coronavirus disease 2019 period, although the difference was not statistically significant (*P*  = .051).

**Conclusion:**

As with many diseases, the diagnosis and treatment of prostate cancer have been adversely affected by the coronavirus disease 2019 pandemic.

**Main Points**

- During the pandemic, it has become necessary to make changes to the prostate cancer treatment guidelines. The prominent approach to the low-risk group has become active surveillance, and alternatives to surgical treatment for the treatment of prostate cancer have gained popularity.
- During the pandemic, the number of radical prostatectomy operations for prostate cancer has decreased significantly.
- The risk of upgrading from biopsy and radical prostatectomy pathology should be kept in mind during the active surveillance of patients.

## Introduction

Prostate cancer (PCa) is the second most common form of cancer in men after lung cancer and is the sixth leading cause of cancer-related death worldwide, with 1.3 million new cases and 359 000 deaths per year.^1^ Age, family history, and black race are the established risk factors for PCa.^2^ With the discovery of prostate-specific antigen (PSA), the disease has become diagnosable early through screening in the asymptomatic period.^3^ Transrectal ultrasonography (TRUS)-guided biopsy is recommended in cases where a digital rectal examination (DRE) finding is suggestive of malignancy or in the presence of abnormal PSA screening results.^4^ For patients diagnosed with localized PCa by TRUS biopsy, the available treatments include radical prostatectomy (RP), radiotherapy, brachytherapy, active surveillance, and watchful waiting.^5^

The viral strain severe acute respiratory syndrome coronavirus-2 (coronavirus disease disease 2019 (COVID-19)) emerged in the Wuhan region of China in late 2019 and developed into a global pandemic that affected millions of people and caused a high number of deaths worldwide.^6^ Physicians and other healthcare workers were reassigned to fight against the pandemic, and intensive care units and other departments were refunctioned for COVID-19 patients. The European Association of Urology has drawn up adaptive guidelines for various situations and to underline the priorities since the viral outbreak. During the COVID-19 pandemic, urological conditions have been classified into 4 priority categories: low priority (can be postponed for 6 months), intermediate priority (can be postponed for 3-4 months), high priority (cannot be postponed for more than 6 weeks), and emergency (cannot be postponed for more than 24 hours).^7^ More than half of men with PCa have comorbidities and are at an advanced age and so are more likely to be affected by COVID-19. It would thus be appropriate to perform such procedures as screening and biopsies during low-risk COVID-19 periods rather than high-risk periods. For localized PCa, treatments such as active surveillance, watchful waiting, and hormonal-radiotherapy are recommended rather than surgery.^8^ It is known from previous studies on localized PCa that delaying RP and radiotherapy causes low-level adverse clinical outcomes.^9,10^ It is also known, however, that metastasis-free survival and disease-specific survival outcomes are poorer in the patient group with a Gleason score of 3 + 4 (moderate risk) than in those with a Gleason score of 3 + 3 (low risk).^11^ Concerning the methods of PCa screening and treatment, it has been reported that all diagnostic and therapeutic procedures used to treat metastatic PCa, except hormonal therapy, decreased in number when compared to the pre-pandemic period.^12^ Our study examines the effects of the COVID-19 pandemic on RP performed as part of localized PCa treatment.

## Materials and Methods

This was a retrospective study. This study included 8 centers in different regions of Turkey. The data of patients who underwent RP for PCa over the 2 years from March 1, 2019, to February 28, 2021, were analyzed retrospectively, with March 1, 2019, to February 28, 2020, denoting the 1-year pre-COVID-19 period and March 1, 2020, to February 28, 2021, denoting the 1-year COVID-19 period. Radical prostatectomy is the most common curative treatment for localized PCa. As in many cancer diseases, surgical treatment is accepted as the gold standard method for the treatment of PCa. The study included 130 (73.9%) patients who underwent RP for localized PCa in the pre-COVID-19 period and 46 (26.1%) patients who underwent RP for localized PCa in the COVID-19 period. Patients older than 18 years were included in the study. Patients with missing file information were excluded from the study.

Data were collected retrospectively from the hospital registration systems. Age, PSA values, DRE findings, history of prostate biopsy, biopsy Gleason score, presence/absence of preoperative imaging and, in case of imaging, lymph node and extraprostatic involvement status, time from biopsy to RP, type of operation, postoperative Gleason score, and pathology data were retrieved from the patient’s files. Magnetic resonance imaging and computed tomography were used in the preoperative imaging of the patients. During the COVID-19 pandemic period, a COVID-19 polymerase chain reaction (PCR) test performed within 72 hours before the operation was requested from all patients. Patients with negative PCR results were operated.

The statistical analysis of the study data was performed in a digital environment using Statistical Package for the Social Sciences software (Version 16.0. Chicago, Ill, USA, SPSS Inc.). The normality of the variables was analyzed with a Kolmogorov–Smirnov test, a Mann–Whitney *U*-test was used for pairwise comparisons of non-normally distributed data, and Pearson’s Chi-square test was used for multiple comparisons. The results were considered statistically significant at *P*  < .05.

The study was approved by Ethical Review Committee (ERC) of Afyonkarahisar Health Science University, Afyonkarahisar, Turkey (No: 2011-KAEK-2/2021/294) and by the institutions in which the research was conducted. This study followed the ethical standards defined in the Declaration of Helsinki. Verbal informed consent was obtained from patients.

## Results

The study included a total of 176 male patients who underwent RP for PCa within a 2-year period. The patients who underwent surgery during the 1-year COVID-19 period were defined as group 1, while those undergoing the operation during the 1-year pre-COVID-19 period were denoted as group 2. The median age of the patients was 65 (43-76) years and 60-69 years for the 25th and 75th percentiles, respectively. The preoperative median PSA value was 9 (2.00-36.00) ng/mL and 6.28-14.79 ng/mL for the 25th and 75th percentiles, respectively. Of the total, 79 (44.9%) patients had no suspicious findings for PCa upon a rectal examination, while 97 (55.1%) patients had findings suggestive of PCa. While 151 (85.8%) patients were diagnosed by the first TRUS-guided biopsy, 25 (14.2%) patients were diagnosed by a repeat TRUS biopsy. The TRUS biopsy pathology was reported as Gleason 3 + 3 in 87 (49.4%) patients, Gleason 3 + 4 in 50 (28.4%) patients, Gleason 4 + 3 in 20 (11.4%) patients, Gleason 4 + 4 in 10 (5.7%) patients, Gleason 4 + 5 in 6 (3.4%) patients, Gleason 5 + 4 in 1 (0.6%) patient, and Gleason 3 + 5 in 2 (1.1%) patients. While 64 (36.4%) patients had prostate tumors in 1 lobe (T2a,b), 112 (63.6%) had tumors in both lobes (T2c). The median of the maximum tumor percentage in the biopsy specimens was 60 (3-100) and 30-90 for the 25th and 75th percentiles, respectively. Prior to RP, 139 (78.98%) patients underwent imaging, while 37 (21.02%) patients did not. Imaging revealed lymph node involvement in 21 (11.93%) patients and extraprostatic involvement in 30 (17.03%) patients. The median time from biopsy to operation was 84 (27-670) days and 59-128 days for the 25th and 75th percentiles, respectively. Open RP was performed in 156 (88.6%) patients, while 20 (11.4%) patients underwent laparoscopic RP. Laparoscopy was performed in 13 patients during the pre-COVID-19 period and in 7 patients during the COVID-19 period. During RP, lymphadenectomy was performed in 95 (54.0%) patients and was not performed in 81 (46%) patients. While lymph node involvement was detected in 22 (12.5%) patients, 73 (41.48%) patients had no such involvement. The median tumor percentage in the RP specimens was 15 (3-95) years and 5.75 and 32.75 for the 25th and 75th percentiles, respectively. The RP Gleason score was 3 + 3 in 48 (27.3%) patients, 3 + 4 in 70 (39.8%) patients, 4 + 3 in 33 (18.8%) patients, 4 + 4 in 6 (3.4%) patients, 4 + 5 in 10 (5.7%) patients, 5 + 4 in 3 (1.7%) patients, 5 + 5 in 1 (0.6%) patient, and 3 + 5 in 5 (2.8%) patients. Extraprostatic involvement was not detected in 112 (63.6%) patients, while 64 (36.4%) patients had extraprostatic involvement. There was no seminal vesicle invasion in 139 (78.98%) patients, while 37 (21.02%) patients had seminal vesicle invasion (Table 1).

A comparison of the patients’ TRUS biopsy and RP pathology Gleason scores revealed a statistically significant difference and upgrading between the biopsy pathology and surgical pathology (*P*  < .001). Of the 87 patients with a biopsy Gleason score of 3 + 3 (low risk), a postoperative pathology was reported as Gleason 3 + 3 in 46 (52.9%) patients, Gleason 3 + 4 in 33 (37.9%) patients, Gleason 4 + 3 in 6 (6.9%) patients, Gleason 4 + 5 in 1 (1.1%) patient, and Gleason 3 + 5 in 1 (1.1%) patient. There was statistically highly significant upgrading after surgery in the low-risk PCa group (*P*  < .001) (Table 2).

Groups 1 and 2 comprised 46 (26.14%) patients and 130 (73.86%) patients, respectively, the difference being statistically significant (*P*  < .001). The median age of the patients was 65 (49-76) years and 60 and 70 years for the 25th and 75th percentiles, respectively, in group 1 and 65 (43-75) years and 61 and 69 years for the 25th and 75th percentiles, respectively, in group 2. There was no statistical difference in age between the groups (*P*  = .789). The pre-biopsy median PSA value was 8.2 (4-29) ng/mL and 5.6-15.25 ng/mL for the 25th and 75th percentiles, respectively, in group 1 and 9.08 (2-36) ng/mL and 6.5-14.77 ng/mL for the 25th and 75th percentiles, respectively, in group 2. There was no statistical difference in the PSA values of the 2 groups (*P*  = .593). When the biopsy Gleason scores were compared, no statistical difference was identified between the groups (*P*  = .158). The median time from biopsy to operation was 84 (30-360) days and 61 and 146 days for the 25th and 75th percentiles, respectively, in group 1 and 85 (27-670) days and 59 and 124 days for the 25th and 75th percentiles, respectively, in group 2. There was no statistical difference in the time from biopsy to operation between the periods (*P*  = .59). While the comparison of RP Gleason scores revealed no difference between the 2 periods (*P*  = .18), there were 8 (17.4%) patients with Gleason 3 + 3 (low risk) in group 1 and 40 (30.8%) patients in group 2. When the 2 periods were compared, the patients with a Gleason score of 3 + 3 were fewer in number in group 1, creating a statistically significant difference (*P*  < .001). In the pre-COVID-19 period, lymph node dissection was performed in 69 (53.1%) patients and lymph node positivity was detected in 20 (15.4%) patients. During the COVID-19 period, lymph node dissection was performed in 24 (52.1%) patients and the lymph nodes were positive in 2 (4.3%) patients. While the rate of lymph node dissection was similar in the 2 periods, there was a statistically insignificant decrease in the number of patients with positive lymph nodes in the COVID-19 period (*P*  = .051). Surgical margin positivity was detected in 26 (20%) patients in the pre-COVID-19 period and in 12 (26.1%) patients in the COVID-19 period. There was no difference in surgical margin positivity between the 2 periods (*P*  = .408) (Table 3).

## Discussion

This study was conducted to examine the effect of the COVID-19 pandemic on RP, and the findings were discussed in the light of literature. Radical prostatectomy operations for PCa were found to decrease in the COVID-19 period. The rate of surgery for Gleason 3 + 3 disease was decreased. A comparison of the biopsy and RP Gleason scores of all study patients revealed a statistically significant upgrading.

Surgical treatment in localized PCa is accepted as the optimum approach, as is the case with several malignant diseases. The number of oncological surgeries has decreased worldwide amid the pandemic as healthcare systems and workers have focused on controlling and treating COVID-19.^6^ Screening, imaging, and biopsies for PCa have had to be postponed during the pandemic.^13^ Although it is known that delays in localized PCa treatment may lead to adverse low-level clinical outcomes,^9,10^ it should be noted that delays in the detection or treatment of PCa may lead to impaired functional outcomes and higher recurrence rates in high-risk PCa.^14^ During the pandemic, the recommended approaches to low-risk PCa were watchful waiting in elderly patients, active surveillance in younger patients, and postponement of treatment for 6-12 months. The treatment of most patients with intermediate-risk PCa can be postponed for 3-6 months without any change in outcomes, and active surveillance may be recommended for eligible patients in this group. Patients who are ineligible for active surveillance should be offered hormonal-radiotherapy, which should also be recommended for patients with high-risk localized PCa and PCa with locally advanced lymph node involvement.^8,15^ Coccolini et al^16^ reported that the operations performed during the pandemic led to an increase in the transmission of COVID-19, with associated increases in morbidity and mortality. Lei et al^17^ reported a mortality rate of 20% and a need for intensive care in 44% of asymptomatic patients who were postoperatively found to be positive for COVID-19. Accordingly, caution should be exercised when deciding upon surgical interventions. Even if patients who are scheduled for surgery have no clinical symptoms or history of contact with COVID-19-positive patients, a COVID-19 test should be performed 48 hours before the operation, and the operation should be performed only after a negative result.^7^ Our study found that the number of RP procedures for localized PCa was highly statistically significantly decreased in the 1-year COVID-19 period when compared to the pre-COVID-19 period. We believe that this decrease may be an outcome of the reduced screenings for PCa, the reduced TRUS biopsies for diagnostic purposes, and the preferred treatment methods, such as active surveillance and hormonal radiotherapy, during the pandemic. There is no clear recommendation on the choice of laparoscopic surgery and open surgery in the COVID-19 pandemic. There are studies showing that the risk of COVID-19 transmission increases or decreases in laparoscopic surgery. Similarly, in our study, the COVID-19 pandemic did not change the preference for open or laparoscopic surgery.^18^

It has been reported that delaying the treatment of patients with low-risk (Gleason 3 + 3) PCa is unlikely to affect the outcome, while delays in treatment may cause adverse outcomes for moderate- or high-risk patients.^19^ We believe that the highly significant decrease in the low-risk patient group when compared to the pre-COVID-19 period can be attributed to the decreased rate of diagnosis of these patients and the transfer of diagnosed patients to other treatment methods, such as active surveillance, watchful waiting, and radiotherapy. In addition, the decrease in the numbers of intermediate and high-risk patients in our study can be attributed to the effect of the pandemic on the application of diagnostic and screening tests.

As PCa is a slow-growing tumor, patients with low-risk PCa can be protected from the complications of unnecessary treatment through active surveillance.^20^ That said, after RP operations for localized PCa, Gleason scores are known to upgrade in a large proportion of patients.^21,22^ When PCa patients with a Gleason score of 7 (moderate risk) and 6 (low risk) were compared in terms of active surveillance, it was observed that patients with a Gleason score of 7 had worse outcomes in terms of metastasis and disease-related survival.^11^ When the biopsy Gleason scores and RP Gleason scores of patients who underwent RP for localized PCa over a 2-year period were compared in the present study, upgrading with a statistically highly significant difference was observed, supporting the findings of previous studies. During the pandemic, patients with low-risk localized PCa should be monitored through active surveillance, while the risk of upgrading should be considered. Such patients should be informed about the situation, and active treatment should be initiated if necessary.

Surgical treatment is recommended as part of a multimodal therapy approach in those with locally advanced disease with lymph node involvement.^23,24^ During the pandemic, it was deemed appropriate to recommend hormonal-radiotherapy rather than surgery as part of the multimodal treatment in this patient group.^8^ Our study’s finding that the rate of patients with positive lymph node involvement statistically insignificantly decreased in the COVID-19 period when compared to the pre-COVID-19 period may be due to the recommendation of hormonal-radiotherapy rather than surgery in these patients or the decreased rate of diagnosis in this patient group.

As the first limitation of our study, we were unable to include patients with inaccessible or missing information due to the retrospective study design; secondly, an unknown number of patients were diagnosed and treated with other methods or monitored by active surveillance during the study period and were also not included in the study; and thirdly there is a lack of knowledge on the situation in other hospitals due to the inclusion of data from only 8 hospitals in Turkey.

The present study shows that RP operations for PCa in Turkey have decreased due to the COVID-19 pandemic, and surgeries performed on low-risk patients were affected more by the situation. While active surveillance is recommended for the low-risk patient group during the pandemic, it should not be forgotten that there is a high rate of upgrading risk in this group of patients.

## Figures and Tables

**Table 1.** Demographic Characteristics of Study Sample

Group 1 (COVID-19 period) (%)		46 (26.1)
Group 2 (Pre-COVID-19) (%)		130 (73.9)
Age, median value (min, max)		65(43-76)
PSA median value (ng/mL) (min, max)		9 (2-36)
Suspicion of Pca in DRE (%)	No	79 (44.9)
Yes	97 (55.1)
TRUS biopsy(%)	First	151 (85.8)
Recurrent	25 (14.2)
Biopsy Gleason score (%)	3 + 3	87 (49.4)
	3 + 4	50 (28.4)
	4 + 3	20 (11.4)
	4 + 4	10 (5.7)
	4 + 5	6 (3.4)
	5 + 4	1 (0.6)
3 + 5	2 (1.1)
Tumor localization (%)	Unilateral	64 (36.4)
Bilateral	112 (63.6)
Median % of tumor in biopsy (min, max)		60 (3-100)
Lymph node in imaging(%)	Not done	37 (21.02)
	Yes	21 (11.93)
No	118 (67.05)
Extraprostatic extension in imaging(%)	Not done	37 (21.02)
	Yes	30 (17.03)
No	109 (61.95)
Median time from biopsy to surgery (min, max)		84 (27-670)
Surgery(%)	Open	156 (88.6)
Laparoscopic	20 (11.4)
Pathology tumor median % (min, max)		15 (3-95)
RP Gleason score (%)	3 + 3	48 (27.3)
	3 + 4	70 (39.8)
	4 + 3	33 (18.8)
	4 + 4	6 (3.4)
	4 + 5	10 (5.7)
	5 + 4	3 (1.7)
	5 + 5	1 (0.6)
3 + 5	5 (2.8)
Lymph node positivity(%)	Not done	81 (46.02)
	Yes	22 (12.5)
No	73 (41.48)
Extraprostatic extension (%)	Yes	64 (36.4)
No	112 (63.6)
Seminal vesicle invasion (%)	Yes	37 (21.02)
No	139 (78.98)

COVID-19, coronavirus disease 2019; PSA, prostate-specific antigen; TRUS, transrectal ultrasonography; RP, radical prostatectomy; DRE, digital rectal examination.

**Table 2.** Comparison of Biopsy and Radical Prostatectomy Gleason Scores

		Radical Prostatectomy Gleason Score								
3 + 3	3 + 4	4 + 3	4 + 4	4 + 5	5 + 4	5 + 5	3 + 5	Total
Biopsy Gleason score	3 + 3	46 (52.9%)	33 (37.9%)	6 (6.9%)	0 (0%)	1 (1.1%)	0 (0%)	0 (0%)	1 (1.1%)	87 (100%)
	3 + 4	1 (2.0%)	31 (62.0%)	13 (26.0%)	0 (0%)	2 (4%)	0 (0%)	0 (0%)	3 (6%)	50 (100%)
	4 + 3	1 (5%)	4 (20%)	12 (60%)	1 (5%)	2 (10%)	0 (0%)	0 (0%)	0 (0%)	20 (100%)
	4 + 4	0 (0%)	2 (20%)	1 (10%)	4 (40%)	2 (20%)	0 (0%)	1 (10%)	0 (0%)	10 (100%)
	4 + 5	0 (0%)	0 (0%)	1 (16.7%)	0 (0%)	3 (50%)	2 (33.3%)	0 (0%)	0 (0%)	6 (100%)
	5 + 4	0 (0%)	0 (0%)	0 (0%)	0 (0%)	0 (0%)	1 (100%)	0 (0%)	0 (0%)	1 (100%)
3 + 5	0 (0%)	0 (0%)	0 (0%)	1 (50%)	0 (0%)	0 (0%)	0 (0%)	1 (50%)	2 (100%)
Total		48 (27.3%)	70 (39.8%)	33 (18.8%)	6 (3.4%)	10 (5.7%)	3 (1.7%)	1 (0.6%)	5 (2.8%)	176 (100%)

**Table 3.** Comparison of Group 1 (COVID-19 Period) and Group 2 (Pre-COVID-19)

		Group 1 (n = 46)	Group 2 (n = 130)	*P*
Age median value (years) (min, max)		65 (49-76)	65 (43-75)	.789
PSA median value (ng/mL) (min, max)		8.2 (4-29)	9.08 (2-36)	.593
Median time between biopsy and operation (days) (min, max)		84 (30-360)	85 (27-670)	.59
TRUS biopsy Gleason score (%)	3 + 3	20 (43.5)	67 (51.5)	.158
	3 + 4	20 (43.5)	30 (23.1)	
	4 + 3	3 (6.5)	17 (13.1)	
	4 + 4	1 (2.2)	9 (6.9)	
	4 + 5	1 (2.2)	5 (3.8)	
	5 + 4	0 (0)	1 (0.8)	
3 + 5	1 (2.2)	1 (0.8)
RP Gleason score (%)	3 + 3	8 (17.4)	40 (30.8)	.18
	3 + 4	24 (52.2)	46 (35.4)	
	4 + 3	9 (19.6)	24 (18.5)	
	4 + 4	2 (4.3)	4 (3.1)	
	4 + 5	1 (2.2)	9 (6.9)	
	5 + 4	0 (0)	3 (2.3)	
	5+5	1 (2.2)	0 (0)	
3 + 5	1 (2.2)	4 (3.1)
Lymph node positivity (%)	Not done	22 (47.8)	61 (46.9)	.051
	No	22 (47.8)	49 (37.7)	
Yes	2 (4.3)	20 (15.4)
Surgical border	Negative	34 (73.9)	104 (80)	.408
Positive	12 (26.1)	26 (20)

COVID-19, coronavirus disease 2019; PSA, prostate-specific antigen; TRUS, transrectal ultrasonography; RP, radical prostatectomy.
